# Pathophysiology of Cerebellar Tremor: The Forward Model-Related Tremor and the Inferior Olive Oscillation-Related Tremor

**DOI:** 10.3389/fneur.2021.694653

**Published:** 2021-06-28

**Authors:** Shinji Kakei, Mario Manto, Hirokazu Tanaka, Hiroshi Mitoma

**Affiliations:** ^1^Department of Anatomy and Physiology, Jissen Women's University, Tokyo, Japan; ^2^Service de Neurologie, Médiathèque Jean Jacquy, CHU-Charleroi, Charleroi, Belgium; ^3^Service des Neurosciences, University of Mons, Mons, Belgium; ^4^Faculty of Information Technology, Tokyo City University, Tokyo, Japan; ^5^Department of Medical Education, Tokyo Medical University, Tokyo, Japan

**Keywords:** tremor, cerebellum, Guillain–Mollaret triangle, predictions, forward model

## Abstract

Lesions in the Guillain–Mollaret (G–M) triangle frequently cause various types of tremors or tremor-like movements. Nevertheless, we know relatively little about their generation mechanisms. The deep cerebellar nuclei (DCN), which is a primary node of the triangle, has two main output paths: the primary excitatory path to the thalamus, the red nucleus (RN), and other brain stem nuclei, and the secondary inhibitory path to the inferior olive (IO). The inhibitory path contributes to the dentato-olivo-cerebellar loop (the short loop), while the excitatory path contributes to the cerebrocerebellar loop (the long loop). We propose a novel hypothesis: each loop contributes to physiologically distinct type of tremors or tremor-like movements. One type of irregular tremor-like movement is caused by a lesion in the cerebrocerebellar loop, which includes the primary path. A lesion in this loop affects the cerebellar forward model and deteriorates its accuracy of prediction and compensation of the feedback delay, resulting in irregular instability of voluntary motor control, i.e., cerebellar ataxia (CA). Therefore, this type of tremor, such as kinetic tremor, is usually associated with other symptoms of CA such as dysmetria. We call this type of tremor forward model-related tremor. The second type of regular tremor appears to be correlated with synchronized oscillation of IO neurons due, at least in animal models, to reduced degrees of freedom in IO activities. The regular burst activity of IO neurons is precisely transmitted along the cerebellocerebral path to the motor cortex before inducing rhythmical reciprocal activities of agonists and antagonists, i.e., tremor. We call this type of tremor IO-oscillation-related tremor. Although this type of regular tremor does not necessarily accompany ataxia, the aberrant IO activities (i.e., aberrant CS activities) may induce secondary maladaptation of cerebellar forward models through aberrant patterns of long-term depression (LTD) and/or long-term potentiation (LTP) of the cerebellar circuitry. Although our hypothesis does not cover all tremors or tremor-like movement disorders, our approach integrates the latest theories of cerebellar physiology and provides explanations how various lesions in or around the G–M triangle results in tremors or tremor-like movements. We propose that tremor results from errors in predictions carried out by the cerebellar circuitry.

## Introduction

The deep cerebellar nuclei (DCN) represent a primary node of the so-called Guillain–Mollaret (G–M) triangle, an anatomical circuit known to play a major role in tremor genesis both in animal models and in human disorders affecting the posterior fossa (1).

Deep cerebellar nuclei have two main output paths: the primary excitatory path to the thalamus, the red nucleus (RN), and other brain stem nuclei, and the secondary inhibitory path to the inferior olive (IO). The inhibitory path contributes to the dentato-olivo-cerebellar loop (we call it the short loop), while the excitatory path contributes to the cerebrocerebellar loop (we call it the long loop).

We propose a hypothesis according to which each loop contributes to physiologically distinct type of tremors or tremor-like movements. One type of irregular tremor-like movement is caused by a lesion in the cerebrocerebellar loop, which includes the primary path. The second type of regular tremor is correlated with synchronized oscillation of IO neurons due to reduced degrees of freedom in IO activities.

## Section I. Phenomenology of Cerebellar Tremors

Cerebellar tremors include diverse phenotypes (2). However, Louis (3) pointed out that, nowadays, “cerebellar tremor is equated exclusively with intention tremor” in an “oversimplified manner” (4). Besides, pathomechanisms underlying essential tremor (ET) have been a focus of debate (2), and thereby roles of IO have likely been overstressed in tremor pathogenesis (5). Due to such a simplification, seminal works by Gordon Holmes appear to be underestimated. This section aims to provide a brief overview of the historical backgrounds and phenomenology of cerebellar tremors.

### Kinetic Tremor and Static Tremor in Holmes' Classic Study

Studies of human cerebellar tremors originate from Holmes' works who carefully examined tremor phenomenology in patients with spatially confined lesions in the cerebellum and described two types of tremors, namely, kinetic tremor and static tremor, in the Croonian lectures given in June 1922 (Table 1). Their clinical phenotypes appear different from those we imagine now from the terminology of kinetic or static. Thus, we cite his original descriptions to elucidate their phenomenology (6, 7). One can read his classic papers in an article of *Cerebellar Classic* (8). It should be acknowledged that these two types of tremors occur concomitantly with deterioration of coordination.

**Table 1:** Clinical features of various forms of tremors described by Holmes: summary of Holmes' Croonian lectures given in June 1922.

	**Holmes' description**	**Static/kinetic**	**Regularity**	**Target oriented**	**Reciprocal muscle activities**	**Contribution of visual feedback**	**Special features**
(1)	Kinetic tremor during motion	Kinetic	Irregular (especially during slow movement)	Yes	Not typical	Yes	Prominent when superior peduncles are damaged. ramya Proximal > distal
(2)	Intention tremor	Kinetic	Irregular	Yes	?	Yes	Tremor associated with disseminated sclerosis. ramya Less sharp than kinetic tremor.
(3)	Static tremor/Gravitational irregular tremor	Static/postural	Irregular	?	No	Yes	Prominent in the extension of both upper limbs. ramya Contribution of fatigue. ramya Proximal joints.
(4)	Static (postural)/Regular oscillatory tremor with reciprocal activities of agonists and antagonists	Static/postural	Regular	Yes	Yes	Yes	Prominent in precise maintenance of the limbs accurately in certain positions. ramya “Terminal tremor”-like tremor. ramya PD rest tremor-like tremor. ramya Easily induced in co-contraction of agonist/antagonist.

#### Kinetic Tremor in Holmes' Classic Study

Holmes described the tremor during active movement (attempts to bring finger from nose to three points in succession or attempts to touch a series of points alternatively) as follows: “At the commencement of the movement the finger or toe may sway from side to side, or the movement may be broken and jerky, especially when performed slowly (Table 1) (1). There is little irregularity as a rule during its course, but in slow and deliberate movements the rate is irregular or discontinuous, or the finger may swing in any plane from the correct line [page 151 in a reference of Cerebellar Classic (8)].” In addition to the irregular and discontinuous sways, he emphasized the association of two additional features. First, terminal tremor (irregular terminal jerks) occurs, associated with hypermetric and hypometric, for example, “in the former case the finger that has shot past its mark is brought back too far and sways or oscillates about its aim until it touches it; in the latter the limb which is arrested before it has reached it is advanced by a series of irregular jerks” (page 151). Second, continuous sways occur at the target. He described that “Even when the finger comes in contact with the patient's nose or other object it may continue to sway from side to side or in the direction of previous movement owing to inability to maintain the attitude steadily” (page 151).

Notably, this kinetic tremor “was less prominent” in most of his cases with local lesions of the cerebellum than in patients with “the primary atrophies (Table 1) (2).” In other words, this type of tremor is prominent in degenerative cerebellar ataxia (CA), suggesting that its developments might be dependent on cerebellar residual functions. Indeed, Holmes hypothesized kinetic tremor, or tremor during active movement, as follows: tremor “naturally results from the irregularities in the rate of muscle contractions, but errors in the range and direction of movement, necessitating correction, are also factors.”

#### Static Tremor in Holmes' Classic Study

Holmes described two types of static tremor.

The first subtype has irregular nature (Table 1) (3). Holmes observed this tremor when his patients extended both upper limbs. He described that “Its oscillations are mostly in the line of gravity, and can be seen on careful inspection to be due to a failure in the tonic contractions of the muscles that maintain the attitude, with the result that the limb falls with gravity and is replaced by voluntary efforts” (page 146). It should be noted that the maintenance of the attitude is a highly voluntary process.

The other subtype is characterized by regular oscillations (Table 1) (4). Holmes described conditions in which this tremor preferably occurs: “Another type of tremor, characterized by more regular oscillations of a limb or some of its segments, occurs when the patient attempts to maintain the limb accurately in certain positions, or in postures necessary for the performance of some act” (page 146). Moreover, “It is usually only in attitudes determined by the tonic contractions of opposing groups of muscles that this *regular* form of tremor develops” (page 146). The lesions of the regular static tremor were ascribed to “the superior peduncles” and “mid-brain lesions that involve these peduncles.”

In summary, the latter type of regular static tremor appears to occur during co-contraction of agonist and antagonist muscles, while the former type of irregular static tremor appears to occur during reciprocal muscle activities for feedback control.

#### After Holmes

For instance, in the “Handbook of Clinical Neurology” published in 1969, Garcin attributed features of kinetic tremor and irregular static tremor in Holmes' classic study to “disturbed continuity of movement” (9). In this regard, he described more clearly features of irregular static tremor as follows: “the static effort of maintaining posture in fact produces tremors to the same extent as does movement,” and “The tremor is more marked when more motor segments are involved, and this explains the difficulty of maintaining immobility in standing or in keeping the arms widely extended” (page 327). He stressed that irregular static tremor is mostly observed in the initial static phase. For example, he described that “at the moment when a hand grasps the glass: when the first clumsy movement is over there may be a few oscillations of pronation and supination occurs, but the patient can grasp the glass without jerking.” This classification of cerebellar tremor by Holmes appears to be used until the 1970s. However, distinction of these two types of tremors are getting rarer in recent textbooks and review articles (2).

### Intention Tremor

Intention tremor was first described by Jean-Martin Charcot. In a well-documented lecture on multiple sclerosis (MS) delivered in 1868, he described the presence of CA in patients with MS, now known as the Charcot's triad (intention tremor, scanning speech, and nystagmus) (10).

A consensus statement of the Movement Disorder Society characterizes features of intention tremor as “amplitude increases during visually guided movements toward a target at the termination of the movement” (11) or “a crescendo increase in tremor occurs as the affected body part approaches its visual target” (12). Furthermore, intention tremor is exaggerated in a visually guided target pursuit task but diminished in a memory-guided task (11, 13). In order to emphasize these pathophysiological features, a term of tremor during target-directed movements has been utilized. Thus, intention tremor can be observed in the finger-to-nose maneuver, which requires precise feedback control. Its frequency is mainly <5 Hz, and “the possibility of a position-specific tremor or a postural tremor produced at the beginning or end of a movement is excluded” (11). Rest tremor is commonly not identified (14). There is a consensus that that intention tremor is caused by a lesion in the cerebellothalamic pathway (12, 14, 15). The lesions are usually in the brainstem in the vicinity of the RN (16) or the posterior thalamus (17, 18). Therefore, another term of cerebellar outflow tremor has also been introduced to stress the neural structure for the genesis of intention tremor (19). In contrast, focal lesions in the cerebellar cortex alone usually do not cause this tremor (20).

In Holmes' classic papers, he described both of his kinetic tremor and regular static tremor occurred in patients with lesions in the superior cerebellar peduncles, suggesting that intention tremor has common features with these Holmes' tremors. Notably, there is a description that “In the tremor that is a prominent feature when the superior peduncles are damaged, the deviations are more abrupt and are terminated more suddenly” (page 151).

### Late-Onset Cerebellar Tremors: “Holmes' Tremor” and Palatal Tremor

The onset of cerebellar tremor after a stroke is diverse, ranging from the day of a stroke to a few years later (14). The above kinetic and static tremors in Holmes' classic study and intention tremor seem to be present in the acute phase. However, the two types of cerebellar tremors also occur characteristically with some delay after the onset of pathologies (21): “Holmes' tremor” and palatal tremor.

#### “Holmes' Tremor”

“Holmes' tremor,” as a modern term, is a rare tremor characterized by the following three features: (1) a concomitant expression of rest tremor and intention tremor, involving the proximal and distal part of the upper limbs with large amplitudes, usually associated with postural tremor; (2) slow frequency, usually <4.5 Hz; and (4) in a case when the preceding lesion (e.g., strokes) is identified, a variable delay (usually 4 weeks−2 years) (11, 14). This unique tremor was previously labeled under rubral tremor or midbrain tremor. However, this tremor is also induced by lesions outside these classic locations. For example, one study of three patients with “Holmes' tremor” following stroke showed that the lesions were located in the superior cerebellar peduncle, midbrain tegmentum, and posterior thalamus (22). To avoid topographic names, therefore, “Holmes' tremor” is now used in honor of his first description (11). Holmes' tremor is frequently accompanied by hypertrophy of the inferior olive nucleus (ION) (23).

#### Palatal Tremor

Palatal tremor is characterized by slow, rhythmic movements of the soft palate (usually, at a frequency of 1–3 Hz) and sometimes of other muscles in the pharynx, larynx, lower face, and trunk (24, 25). Palatal tremor comprises idiopathic and symptomatic types. The causes of symptomatic palatal tremor include stroke, trauma, MS, Behçet's disease, and encephalitis (24), and the most common causes are strokes in the brainstem and the cerebellum (24). The symptomatic palatal tremor usually develops some time (1–49 weeks) after the lesion onset (26), which is associated with cerebellar symptoms (25) and hypertrophy of ION (24, 25).

Taken together, the late-onset nature and the associated ION hypertrophy suggest underlying secondary and compensatory pathological mechanisms in “Holmes' tremor” and palatal tremor (21). The hypertrophy of ION is usually observed as a high signal on T2- or proton density-weighted MR image with the enlargement (24, 25).

### Essential Tremor

According to a consensus statement of the Movement Disorder Society, ET is defined by bilateral, largely symmetric postural, or kinetic tremor, at the frequency of 4–12 Hz, involving hands and forearm, with or without head tremor and tremor in other locations (11, 12). The primary clinical phenotype is the postural tremor of the hands (11). The tremor generally persists, although the amplitude fluctuates (11). The tremor may or may not produce disability (11); however, ET is progressive in nature (27). Concomitant manifestation of intention tremor and rest tremor is observed in 50 and 20% of the patients, respectively (27). Due to the heterogeneity, it is proposed that ET comprises a family of diseases rather than a single entity (27). In other words, ET is overlapping clinical phenotypes.

In the 2018 statement, the notion of ET plus was introduced to include patients with neurological signs of uncertain relationship to tremor (i.e., “soft neurological signs”). Notably, soft neurological signs include cerebellar symptoms such as a mild degree of ataxic gait, oculomotor deficits, and impaired motor timing (27). Due to the clinical heterogeneity, Louis et al. (28) proposed that ET may represent a family of diseases rather than a single clinical–pathological entity (28). Our current understanding of the mechanisms behind ET has evolved quickly, thanks to the works of Louis' group with the elucidation that cerebellar cortex shows abnormal features in postmortem material (29). These authors have shown abnormalities in Purkinje cells (PCs: axonal swellings, swellings in and regression of the PC dendritic arbor, and PC death), basket cells, and climbing fibers in individuals with ET.

In conclusion, cerebellar tremors gather various phenotypes (Table 2). Two clinical features will be summarized:

1. Tremor is generally defined as the “involuntary, rhythmic, oscillatory movement of a body part” (11, 12). However, the irregularity in cycle and amplitude is evident in kinetic tremor and irregular static tremor in Holmes' classic study, and sometimes in intention tremor, compared with other types of cerebellar tremors.
2. In the condition of “Holmes' tremor” and ET, plural pathophysiological mechanisms appear to contribute to their phenotypes of tremor either concomitantly or with the lapse of time.

**Table 2:** Phenotypes in cerebellar tremors.

**Type of cerebellar tremor**	**Phenomenology**	**Responsible region**
Kinetic tremor in Holmes' classic study	• Irregular and discontinuous sways • Sometimes marked at the beginning of the movement	The cerebellum (probably destruction of the cerebellar cortex and/or the white matter)
Static tremor in Holmes' classic study	• Subtype 1: Irregular oscillation in the extension of upper limbs during the maintenance of the limb against gravity • Subtype II: Regular oscillations of a limb or some of its segments during maintenance of the limb accurately in certain positions	The cerebellum (probably destruction of the cerebellar cortex and/or the white matter)
Intention tremor	• Amplitude increase during visually guided movements toward a target at the movement termination	The dentato-rubro-thalamic tract
“Holmes' tremor”	• Concomitant expression of rest tremor^*^ and intention tremor with/without postural tremor^*^ • Slow frequency, usually <4.5 Hz • Late onset of pathologies	Superior peduncle, midbrain tegmentum, and posterior thalamus
Palatal tremor	• Rhythmic movements of the soft palate • Late onset of pathologies	The brainstem and the cerebellum
Essential tremor	• Bilateral, largely symmetric postural tremor or kinetic tremor^*^ • Involving hands and forearm, with or without head tremor and tremor in other locations	Cerebellar cortex

*Kinetic tremor, tremor occurring during any voluntary movement; postural tremor, tremor present while voluntarily maintaining a position against gravity; rest tremor, tremor that occurs in a body part that is not voluntarily activated and is completely supported against gravity*.

* *Definition by Consensus Statement of the Movement Disorder Society on Tremor (11)*.

From physiological and control engineering points of view, difference in regularity and voluntariness strongly suggests contribution of distinct control mechanisms. In addition, difference in onset also suggests distinct pathomechanisms to be factored in. Overall, the three factors, i.e., regularity, voluntariness, and onset, may be key clues for understanding pathophysiology of diverse cerebellar tremors. We will address this issue in section Physiological Backgrounds of Two Types of Cerebellar Tremors.

## Section II. Physiological Backgrounds of Two types of Cerebellar Tremors

In the previous section, we traced the historical backgrounds and phenomenology of cerebellar tremors as far back as the original descriptions by Holmes (6, 7). We realized that various phenotypes of “cerebellar tremors” may contain two distinct conditions: involuntary regular tremors and voluntary irregular tremors (or more precisely, tremor-like movements), and each condition may be related to distinct pathology of distinct neuron circuitries. In this section, we will address the two tremor generation mechanisms based on recent physiological, morphological, and clinical findings.

### Two Loop Circuitries in the Dentato-Rubro-Olivary (Guillain–Mollaret) Triangle and Their Functions

**Figure 1:**
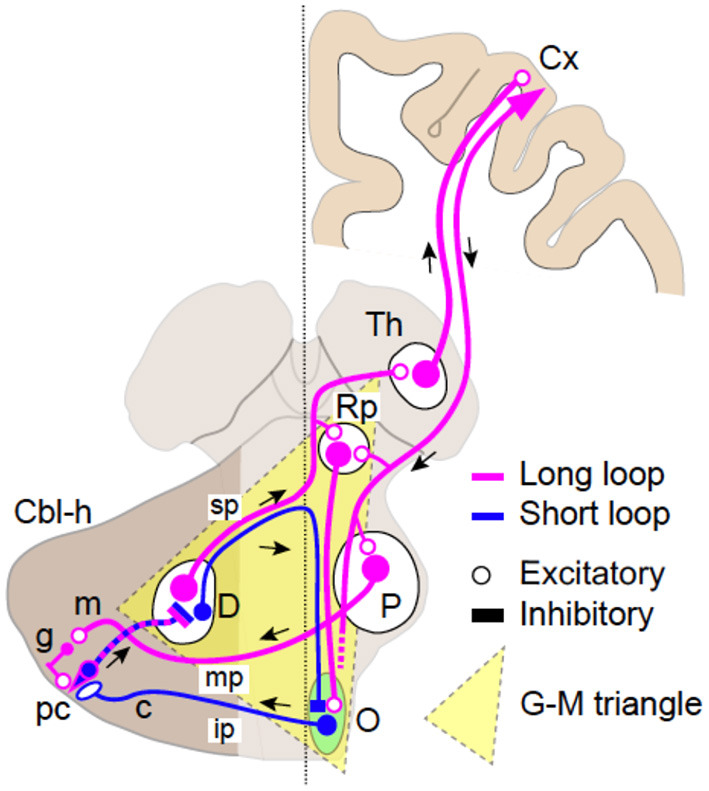
Schematics of the two loop circuits in the Guillain–Mollaret triangle. The dentato-olivo-cerebellar loop (short loop, blue) and the cerebrocerebellar loop (long loop, magenta). Smaller GABAergic (inhibitory) cells in the dentate nucleus (D) pass through the superior cerebellar peduncle (SCP) (sp), cross the midline, and project directly to the contralateral inferior olivary nucleus (O). Efferent fibers from IO pass through the inferior cerebellar peduncle (ip) and project to Purkinje cells (PC, pc) in the contralateral cerebellar hemisphere (Cbl-h). PCs then project to DN cells to close the loop. The long loop is almost identical to the cerebrocerebellar loop. Larger excitatory DN cells pass through (SCP, sp), cross the midline, and project to the contralateral parvocellular red nucleus (RNp), and the thalamus (Th) with collaterals. Thalamocortical neurons relay the cerebellar inputs to various cortical areas (Cx). The return path to the cerebellum is the cortico-ponto-cerebellar tract, which originates from a various parts of the cerebral cortex. The corticofugal axons project directly to the PN (P) and finally arrive at the contralateral cerebellar hemisphere (Cbl-h) as mossy fibers (MFs) via the middle cerebellar peduncle (mp) to close the loop.

**Figure 2:**
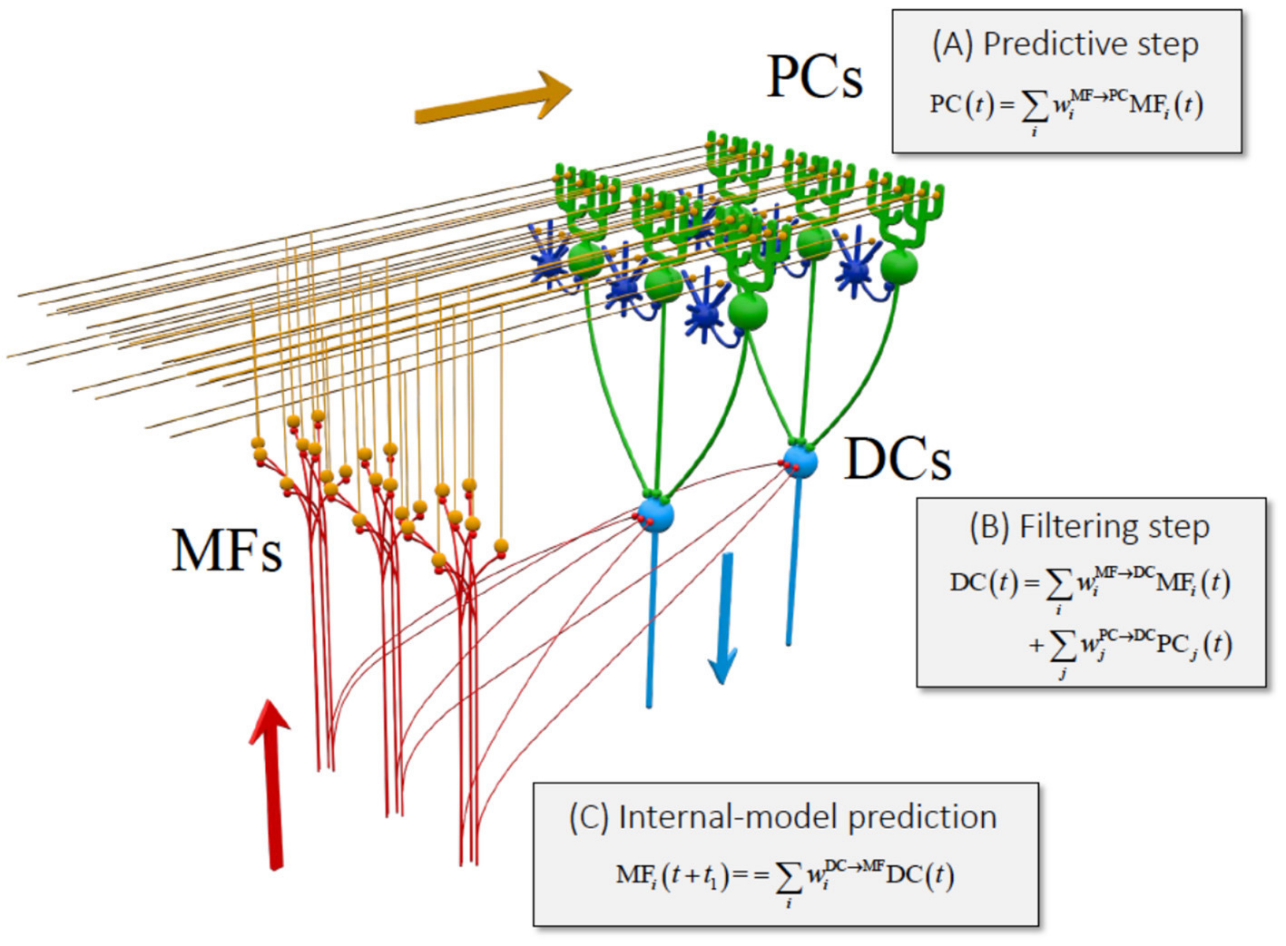
Equivalence of the cerebrocerebellar circuitry to a Kalman filter [reproduced with permission from Tanaka et al. (63)]. Schematic of the Kalman filter model of the cerebrocerebellum overlaid on the cerebellar circuit. MF, mossy fiber (red); PC, Purkinje cell (green); DC, dentate cell (light blue). Granule cells (orange) and inhibitory interneurons (blue) that are not analyzed in this work are included to show the basic structure of the cerebellar neuron circuitry. Three stages of linear computation obtained in our analysis are accompanied with the three types of computation of Kalman filter explained in the text. Reproduced from Tanaka et al. (63) under CC-BY license.

It has long been established that patients with lesions in or in the vicinity of the G–M triangle (Figure 1) frequently show various types of tremors or tremor-like movements (14). Previous studies established that the G–M triangle contains two distinct loop circuitries: (1) the dentato-olivo-cerebellar loop (we call it *the short loop*, Figure 1) and (2) the cerebrocerebellar loop (we call it *the long loop*, Figure 1).

#### Anatomy of the Long Loop

The long loop is almost identical to the cerebrocerebellar loop (30–32). Larger excitatory dentate nucleus (DN) cells, after passing through SCP (Figure 1, sp) and crossing the midline, project to the contralateral RNp and the thalamus (Figure 1, Th) with collaterals. Thalamocortical neurons relays the cerebellar inputs to various cortical areas (Figure 1, Cx). The return path to the cerebellum is the cortico-ponto-cerebellar tract, which originates from various parts of the cerebral cortex (30–32). The corticofugal axons project directly to the pontine nuclei (PN, Figure 1, P) and finally arrive at the contralateral cerebellar hemisphere (Figure 1, Cbl-h) as mossy fibers (MFs) via the middle cerebellar peduncle (Figure 1, mp) to close the loop (30, 31).

#### Physiological Operation of the Short Loop

The DN contains two distinct types of neurons. Larger excitatory neurons project to the parvocellular part of the red nucleus (RNp) and the thalamus (Th), while smaller inhibitory (GABAergic) neurons project directly to the IO to inhibit IO neurons (33). The GABAergic terminals in IO are concentrated around gap junctions between the IO neurons (34) and reduces their conductance, thereby reducing synchronous activities of the IO neurons (35). On the other hand, IO neurons also receive excitatory inputs from PNp (35–37). The excitatory terminals are concentrated around the gap junctions and are presumed to facilitate synchronous activities of the IO neurons (34, 35). In summary, the IO neurons receive two distinct types of inputs; one facilitates, and the other suppresses synchronous activities of IO neurons.

##### A Putative Servo-Like Mechanism to Limit the Synchrony of IO Neurons

In physiological conditions, the inhibitory input from DN and the excitatory input from RNp to the gap junctions between the IO neurons appear to be balanced. For instance, when DN cells get more active, the direct inhibition from DN to IO increases, while the disynaptic excitatory input from RNp to IO also increases concomitantly. In contrast, when DN cells get inhibited, the direct inhibition from DN to IO decreases (i.e., disinhibition), while the disynaptic excitation from RNp to IO decreases concomitantly. In summary, regardless of the alteration of output from DN, modulations of inhibitory and excitatory inputs to IO appear to cancel each other. Overall, the synchrony between IO neurons appears to be limited within a certain range in physiological conditions with this servo-like mechanism.

#### Physiological Operation of the Long Loop: the Cerebrocerebellum as a Site of Forward Models

One critical problem in biological motor control is that afferent sensory signals have inevitable temporal delays in reaching the central nervous system. In other words, the brain always observes “the past” of its own body and environments. A visual signal, for instance, arrive at the primary visual cortex about 30 ms later and at the parietal cortex about 80 ms later than an onset of the signal (38). Among the factors contributing to the feedback delay, such as a synaptic delay or an electro-mechanical delay, the dominant factor is the nerve conduction delay, ranging about 10 ms for a shrew to about 100 ms for an elephant. Sensory delays are comparable to typical time scales of rapid movements and hence not negligible.

The delay in sensory feedback is problematic not only in sensing the body and the environments but also in controlling the body. It is well known in control engineering that feedback control based on a previous state causes oscillatory and unstable movements if the delay in feedback control is of the order of or larger than a time constant of a controlled plant (39). The delays in visual feedback are comparable to the movement time of rapid reaching movement of the upper limb (about a few hundred milliseconds) and of saccadic eye movements (typically <50 ms). Therefore, in biological motor control, feedback control based on delayed sensory signals would result in unstable movements. Nonetheless, animals can perform a fast movement without losing its stability. Biological motor control must be equipped with a mechanism to compensate the sensory delay for a fast and stable movement.

One mechanism proposed to cope with the delay in sensory feedback is to compute a future state of the body based on a current estimate of the body and an efferent signal of motor control. This predictive computation internally emulates or models an actual movement of the body by essentially solving an equation of motion of the body forward in time, thereby known as an internal forward model (40, 41). An internal forward model predicts the state of the body time by time that is then used by a feedback controller, thereby allowing fast and stable movements. The feedback control based on the prediction of internal forward model is called internal feedback. There are lines of evidence supporting the hypothesis of predictive forward model and internal feedback from neuroimaging studies (42, 43), non-invasive stimulation studies (44, 45), and psychophysics studies (46–48) in human.

Previous studies repeatedly suggested the cerebrocerebellum as a potential site of the forward model based on neuroanatomical data and clinical observations [e.g., (39, 49–52)]. A forward model requires two distinct inputs: (a) a set of sensory feedback signals, which are necessary to update the forward model and (b) the copy of descending motor commands. The two inputs are integrated in the forward model to generate the state estimate. In fact, the cerebellum receives both of these inputs. It receives inputs from cortical motor areas via the PN (53, 54), and these inputs represent the efference copy of descending motor commands (55–57). The cerebellum also receives somatosensory inputs directly from the ascending spinocerebellar tracts and indirectly via brain stem nuclei, such as the cuneate nucleus or the lateral reticular nucleus. These sensory inputs may provide an update on the state to be estimated. The above argument may appear to support the cerebellar forward model hypothesis. However, in reality, it is on insufficient grounds because the two lines of inputs are primarily separate in the cerebellar cortex. The MF inputs from the cortical motor areas (via PN) distribute mainly in the hemispheric (i.e., lateral) part (58), while the sensory MF inputs from the spinal cord or the brain stem nuclei distribute in more rostral and medial part (the anterior lobe and the intermediate zone) [e.g., (59)] of the cerebellar cortex. Therefore, one may expect a convergence of the two MF inputs only in a minor part of the intermediate zone. Unfortunately, the simple summation of the two MF inputs is not consistent with their asymmetric roles in the forward model. The efference copy plays an essential role in a state prediction, while the sensory input plays a critical role in an update of the prediction, as will be discussed later.

As for the output from a forward model, we expect it to correlate with the future state of the motor apparatus (39). In principle, we should examine the output from the cerebrocerebellum in the DN because it is the sole output node from the cerebrocerebellum. Nevertheless, previous studies tried to address this issue by analyzing the PC activities. Note that the PCs' activity represents an intermediate representation of the cerebellar circuitry and is not suitable for characterizing the output of a forward model. In this regard, few studies are eligible to discuss the output of the cerebellar forward models (60–62).

##### System Identification of the Transformation in the Cerebrocerebellum—Its Similarity to Kalman Filter

**Figure 3:**
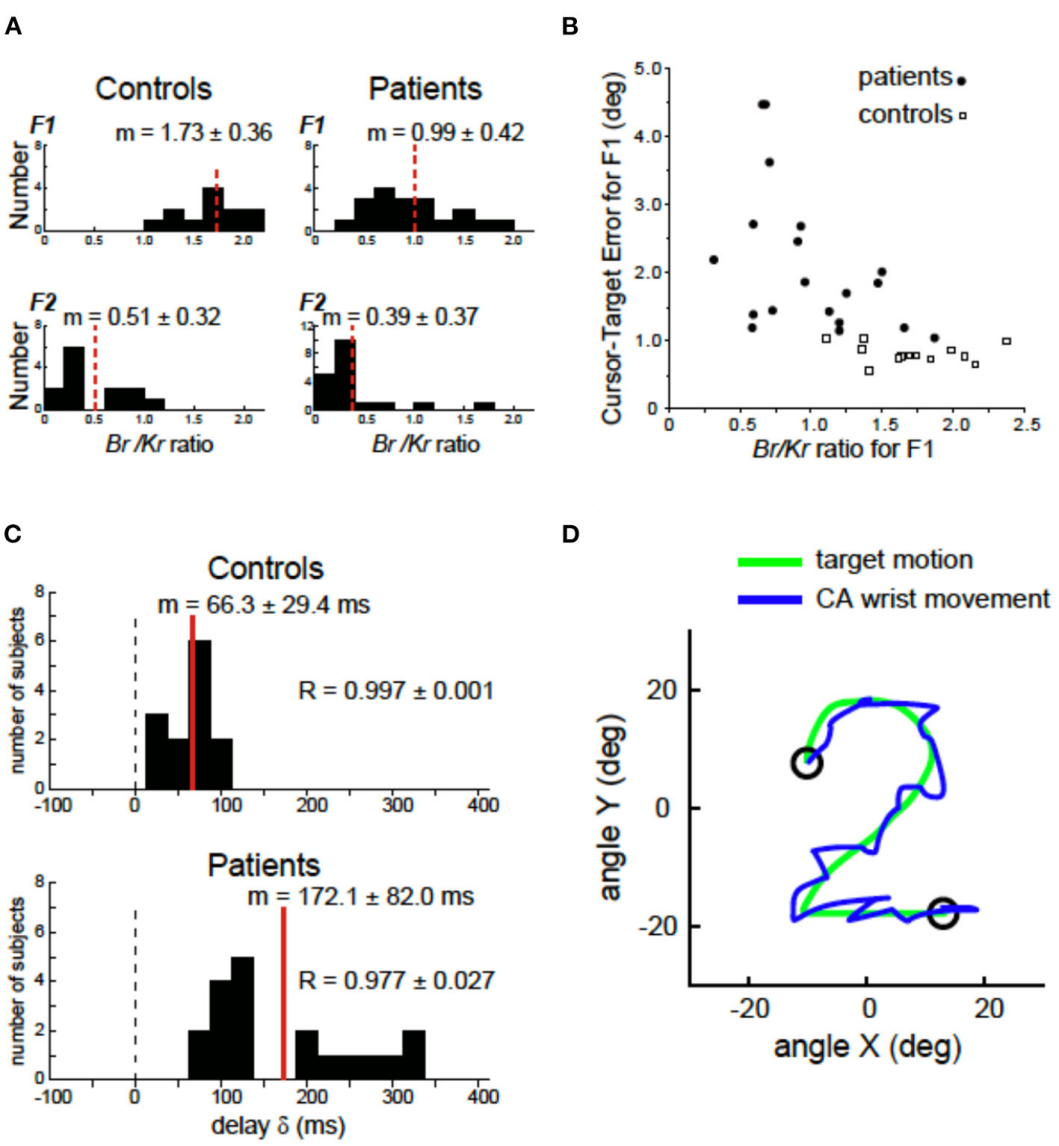
Deficits of forward models in patients with cerebellar ataxia (CA). **(A)** Comparison of the Br/Kr ratios that represents recipe of the motor commands for the F1 and F2 components between the controls and the cerebellar patients. Controls: Br/Kr ratios of the control subjects for the F1 component (top) and the F2 component (bottom) (*n* = 13). Note the highly significant difference between the two components. Patients: Br/Kr ratios of the patients for the F1 (top) and the F2 (bottom) components (*n* = 19). Note the selective decrease in Br/Kr ratios for the F1 component in the patients. **(B)** Correlation between the Br/Kr ratios for F1 component and cursor–target error for F1 (F1 error, in short). The F1 error is defined as an average error between the target motion and the F1 component of the movement. Note the negative correlation. **(C)** Delay of the predictive (F1) component of the movement relative to the target motion calculated with a cross-correlation analysis for controls (*n* = 13) and patients (*n* = 19). **(D)** A highly ataxic wrist movement of a CA patient. Note the irregular tremor-like movement trajectory. Adapted from Kakei et al. (69) under CC-BY license.

If the cerebrocerebellum functions as a forward model, it is expected that the current output from DN should contain predictive information about the future MF input. Therefore, in our previous study (63, 64), we examined the relationship between activities of MFs (cerebellar inputs), PCs (intermediate representation), and DNCs (cerebellar outputs) (**Figure 3**). Briefly, we demonstrated that the activities of individual PCs were reconstructed precisely as a weighted sum of those of MFs. Similarly, the activities of individual DNCs were reconstructed strictly as a weighted sum of those of PCs and MFs. We further proved that the activities of DNCs contained predictive information about future MF inputs (63, 64). Namely, the output from the cerebrocerebellum is capable of predicting 200 ms into the future to compensate for the delay of sensory feedback. We finally note that the linear relationship between MF, PC, and DNC activities resemble an optimal linear estimator known as the Kalman filter [(63–65)].

The functional similarity of the cerebellum to the Kalman filter has already been suggested in some previous studies. Most notably, Paulin (66, 67) indicated that the cerebellum could be a neural analog of a Kalman filter. Droulez and Cornílleau-Pérèz (68) drew attention to the relevance of multisensory integration in the moving organism to the Kalman filter. Nevertheless, the suggested analogy was only at the functional level and totally lacked correspondence to the cerebellar network. In our study, we demonstrated the three computational steps in the cerebellar circuit that are compatible with the Kalman filter (63, 64) (Figure 2): (1) the PCs compute a predictive state from a current estimate conveyed by the MFs (prediction step); (2) the DNCs combine the predicted state from the PCs and sensory feedback from the MFs (Filtering step); and (4) the DNCs represent future activities of MFs (cerebellar prediction).

Overall, the cerebellum appears to perform not only an internal-forward-model prediction but also an optimal integration of a predicted state and sensory feedback signals, in a way that is equivalent to Kalman filter as demonstrated in Tanaka et al. (63, 64) (Figure 2).

#### Interaction Between the Two Loops

It should be noted that the two loops are not independent to each other as clearly depicted in Figure 1. First, they share the same PCs in the hemispheric part of the cerebellar cortex. Second, the long loop has a side path to modulate activities of IO cells through RNp. Therefore, the two loops are interactive and dependent to each other. An unstable loop may therefore impact on the physiological behavior of the second loop. Abnormal discharges may emerge from altered PCs (see the example of ET), and this will impact on both loops.

### Generation of Two Types of Tremors

We underline that both loops are designed to avoid tremor or instability as described above. Indeed, the short loop has a neural mechanism to avoid synchronous discharges of IO neurons, while the long loop has evolved to function as a forward model to avoid instability of control. Nevertheless, in pathological conditions, each safety mechanism fails, resulting in the generation of a characteristic type of tremor.

#### Failure of the Short Loop Results in Regular Oscillatory Tremors

As reviewed in section Phenomenology of cerebellar tremors, the modern definition of the term “tremor” is “the involuntary, rhythmic, oscillatory movement of a body part” (11, 12). Naturally, a number of previous studies, both basic and clinical, addressed the location of the oscillator. There is a consensus that IO plays an essential role in the generation of the regular tremors (35, 70, 71). For instance, harmaline-induced tremor in rodents has been extensively used as an animal model for ET. Cheng et al. (72) made a subcutaneous injection of harmaline hydrochloride (20 mg/kg) in mice and then videotaped the responses. Regular action and postural tremors in the mouse began no more than 5 min after harmaline injection and peaked at approximately 30 min. The forelimb tremor was postural or action tremor, similar to that observed in ET. In these model animals, a large population of IO neurons appear to discharge in synchrony and rhythmically (73–75), thereby inducing synchronized complex spikes (CSs) of Purkinje cells. Then, the synchronized CSs ignite synchronized rebound excitation of DN cells (71, 76), and the cerebellar output finally induces, through the thalamocortical pathway, rhythmical and reciprocal discharges of agonists and antagonists muscles, i.e., tremor. As described in *Physiological operation of the short loop*, there is a mechanism to avoid synchronous discharges of IO neurons in physiological conditions. Nevertheless, in pathological conditions and for specific posture and/or movement, IO neurons are somehow switched into a synchronization mode to induce rhythmical discharges, resulting in regular tremors. We infer that involuntary and regular tremors, such as static tremor described by Holmes (6, 7), rest tremor and postural tremor of “Holmes' tremor,” and ET, are likely to depend on the same mechanism described above. We also infer that “Holmes' tremor” and palatal tremor depend on the same mechanism, although the efferent pathway of the palatal tremor appears to spare the Vim nucleus of the thalamus because Vim thalamotomy is ineffective to palatal tremor, while it is effective to “Holmes' tremor” (77).

#### Generation of Irregular Tremor-Like Movement and Its Relevance to the Forward Model Hypothesis of the Cerebellum

Not all tremors or tremor-like movements are regular or oscillatory (see section Phenomenology of Cerebellar Tremors) as noted by Holmes himself (6, 7). The irregularity in cycle and amplitude is crucial because it strongly suggests different generation mechanisms from that of the regular tremors described above. Moreover, it should be noted that the irregularity appears during voluntary movement, as exemplified in their names “kinetic” or “intention.” Here, we explain the irregularity (i.e., kinetic tremor in Holmes' classic study and intention tremor) as malfunction of the cerebellar forward model.

In our previous study (69), we demonstrated a clinical evidence that supported the cerebellar forward model hypothesis [e.g., (44, 51)]. A series of studies from our group confirmed the impaired predictive control in movements of patients with degenerative CA. We first decomposed the muscle activities for the wrist movement into a low-frequency ( ≤ 0.5 Hz) component (F1) and a high-frequency (>0.5 Hz) component (F2), each of which represented the predictive control and the feedback correction, respectively (69). Then, for each component, we identified a recipe of muscle activities by analyzing a relationship between the muscle tension and movement kinematics [the wrist angle θ(*t*) and the wrist angular velocity $\overset{∙}{\theta }\left(t\right)$] weighted by the coefficients of *K*_*r*_ (the elastic term) and *B*_*r*_ (the viscous term) (69, 78–80). Importantly, the ratio of *B*_*r*_*/K*_*r*_ characterized the recipe of muscle activities for the predictive and corrective components. In control subjects, the *B*_*r*_*/K*_*r*_ ratio for the predictive (F1) component demonstrated a higher value (Figure 3A), suggesting the velocity control dominance. On the other hand, the *B*_*r*_*/K*_*r*_ ratio for the corrective (F2) component demonstrated a much smaller value (Figure 3A), suggesting the role of F2 component in correction of positional errors (69). In contrast, CAs showed a selective decrease in the *B*_*r*_*/K*_*r*_ ratio for the predictive (F1) component (Figure 3A), suggesting poor recruitment of the predictive velocity control and compensatory dependence on the position-dependent pursuit (69). The loss of component-specific differences in the *B*_*r*_*/K*_*r*_ ratio suggests impairment of predictive control in CA. Indeed, the decrease in B_r_/K_r_ ratio in CA correlated with the increase in error in the predictive (F1) movement (Figure 3B) (69). Another critical difference between the control and CA was the increased delay of the predictive (F1) component in CA (Figure 3C). In the control subjects, the predictive (F1) movement lagged the target motion only by 66 ms, which was too small to be a visual feedback delay (i.e., a proof of prediction) (69). In contrast, in patients with CA, the delay increased by more than 100 ms, as much as 172 ms. The increased delay is comparable to a visual feedback delay, demonstrating lack of compensation of feedback delay in CA patients. In summary, ataxic movements are consistent with an impairment of a forward model in terms of accuracy and delay of state prediction. As mentioned already, the delay in prediction alone provides instability in control of goal-directed movement. Moreover, the increase in prediction error makes the oscillatory movement irregular because it makes uncertainty of each corrective (i.e., feedback) movement unreliable due to increased uncertainty of both current and future states. The residual errors trigger a chain of irregular corrective movements around the target trajectory (Figure 3D, CA wrist movement). Note that the chain of corrective movements (i.e., the tremor-like movement in Figure 3D) is voluntary in nature, although it must be far from what CA patients intended to do.

As demonstrated in Figure 1, the long loop could be disrupted at any point along the loop. In addition, the disruption may vary from a partial one to a complete one. In case of a complete disruption, malfunction of the forward model may be irreversible, and the resultant irregular tremor must be severe and persisting because the cerebellar reserve (81) is unavailable. In contrast, in case of a partial disruption, the initial irregular tremor may recover partially or completely depending on the level of compensation with the cerebellar reserve. For instance, Sasaki and his colleagues made cerebellar hemispherectomy in monkeys trained for skilled hand movements and observed CA for many months (82, 83). When the lesion involved both DN and interpositus nuclei (IN), the monkeys revealed typical cerebellar symptoms, hypotonia, asthenia, awkwardness, dysmetria, and kinetic and/or static tremor. These symptoms lasted for several months until the animals were sacrificed. However, in the cases in which the lesion involved DN but spared IN, the symptoms disappeared in a few weeks.

These studies suggest that cerebellar reserve is damaged more severely in a lesion in the SCP than in a lesion in the cerebellar hemisphere. Thus, tremor in the former lesion (e.g., intention tremor) develops more irregular and abrupt natures compared with tremor in the latter lesion (e.g., kinetic tremor in Holmes' classic study). In this regard, this type of irregular tremor may disappear in a short period when the cerebellar reserve is available, as typically seen in patients with a localized cerebellar stroke.

### Impairments in “G–M Triangle”

#### Disruptions of the Two Loops in the “G–M Triangle”

The G–M triangle includes vital parts of the long loop and the short loop. In particular, both loops are packed into the same bundle in SCP (Figure 1, sp). On the other hand, after crossing the midline, SCP is divided into the ascending branch and the descending branch (84). The ascending branch mainly contains thicker excitatory fibers from DN, while the descending branch mainly contains finer inhibitory fibers from DN (34). Therefore, a focal lesion of SCP or a large lesion in the G–M triangle may disrupt both loops. On the other hand, a localized lesion of the ascending branch or the descending branch may disrupt the long loop or the short loop separately.

For instance, a selective disruption of the long loop disorganizes the online operation of cerebellar forward model and leads to manifestation of irregular tremors, including kinetic tremor in Holmes' classic study and intention tremor, when the dysfunction exceeds a threshold. We also hypothesize that the disruption of the short loop (i.e., removal of inhibition on the gap junctions between IO neurons) shifts IO activities toward the synchronous mode like a local injection of bicuculine into IO (85) to cause regular tremors such as regular postural tremor in Holmes' classic study.

**Figure 4:**
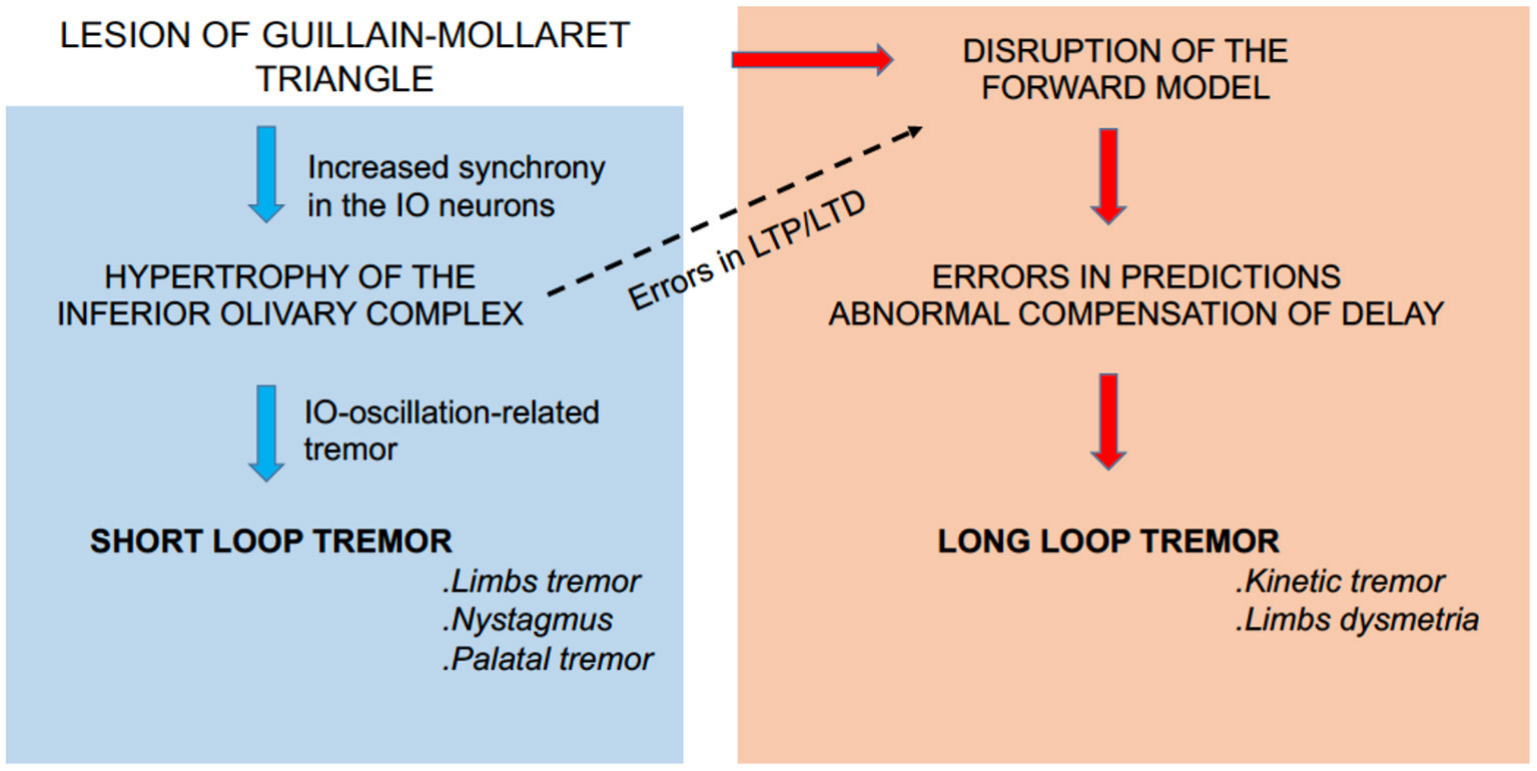
Summary diagram. A lesion in the G–M triangle may well-disrupt the short loop (left panel) and the long loop (right panel) to cause the diverse types of tremors. In addition, the aberrant activities in the short loop (i.e., aberrant complex spike activities) may induce *secondary* maladaptation of cerebellar forward models through aberrant patterns of LTD and/or LTP of the cerebellar circuitry (dashed arrow).

It has been a focus of debate why “Holmes' tremor” exhibits diverse types of tremors (i.e., rest, postural, and intention tremors) after a period of time. “Holmes' tremor” (midbrain tremor) was previously called cerebellar outflow tremor, whose causal lesions include SCP, midbrain tegmentum, or posterior thalamus. These foci are aligned on the dentato-thalamic (DN-Th) tract and are in or close to the G–M triangle (Figure 1). A lesion in the G–M triangle may well disrupt the two loops in a complicated manner, causing the diverse types of tremors (Figure 4).

#### Reorganization and Maladaptation in the G–M Triangle

##### Reorganization in the Short Loop

Emergence of regular rest or postural tremors in “Holmes' tremor” needs several weeks or longer (usually 4 weeks−2 years) after disruption of the short loop. The longer latent period may correspond to the time required for synaptic reorganization around the gap junctions of IO neurons, i.e., reduction or disappearance of inhibitory terminals and concomitant sprouting of excitatory terminals (86, 87). However, this hypothesis does not exclude possibility of regular tremors during acute phases (14). For instance, the above-mentioned harmaline-induced tremor model clearly suggests the existence of a switch to ignite regular tremors without chronic reorganizations of neuron circuitries.

##### Induction of Maladaptation Caused by Regular Tremors

The regular tremor is accompanied by abnormal synchronized IO activities. The aberrant IO activities (i.e., aberrant CS activities) may induce secondary maladaptation of cerebellar forward models through aberrant patterns of LTD and/or LTP of the cerebellar circuitry (Figure 4, dashed arrow). The problem may be twofold. First, during a regular tremor, average CS activities (>4 Hz) are much higher than normal levels of CS activities (~1 Hz). Therefore, CS activities are corrupted by increased noise (i.e., low S/N ratio) during regular tremors. Second, Hoang et al. (85) recently found that high coupling strengths of IO neurons induce their synchronous firing and decrease the amount of information encoded by firing dynamics of IO neurons. The two mechanisms may gradually deteriorate the forward model and increase its prediction error, resulting in irregular tremor. In this regard, it may be possible to explain the intention tremor of “Holmes' tremor” with this mechanism.

In conclusion, it is important to note that in “Holmes' tremor,” or more generally tremors induced by lesions in the G–M triangle, disruptions of the two loops coexist and induce the regular and irregular types of tremors in various combinations depending on the location and size of the lesion. In addition, the complex pathological condition is further prone to secondary changes such as reorganization and maladaptation.

### Consideration of Neuroimaging Studies

Our proposal of a dual pathogenesis will now require an in-depth multimodal assessment to establish how it can be translated into a direct clinical practice. This ambitious goal will likely remain a highly challenging task. For the time being, let us conclude this manuscript with a brief consideration of neuroimaging studies because it allows to assess the morphological and functional aspects in cerebellar tremor patients. Structural imaging by MRI provides insights for focal or diffuse anatomical lesions, complemented in particular by diffusion imaging (DTI), fMRI, and assessment of metabolic brain networks (88, 89). Diffusion tractography shows the neuronal connections in the brain and allows to draw conclusions in terms of deafferentation following a focal lesion such as a stroke and infer on remote effects of this connection.

One typical example was provided by Seidel et al. who reported the case of a 20-year-old patient with right-sided Holmes' tremor 9 months after a midbrain/pontine hemorrhage (90). Tractography demonstrated a reduced fiber connectivity of the superior and middle cerebellar peduncles on the lesioned side. The hemorrhage affected the RN directly and impacted on nigro-striatal projections and the cortico-rubro-cerebellar loop, underlining that tremor was probably due to a deafferentation mechanism (88). These findings are coincident with the present proposal of reorganizations in the short loop (see section Reorganization and Maladaptation in the G–M Triangle). Tractography has been used successfully to target the dentato-rubro-thalamic tract to plan the implantation of electrodes for deep brain stimulation in combination with traditional landmark-based targeting techniques (91).

In ET, a functional disconnection of dentate nuclei with cortical, subcortical, and cerebellar areas has been demonstrated recently (92). Changes in the cerebellum positively correlated with tremor amplitude, in contrast with changes in the bilateral thalamus that negatively correlated with tremor amplitude. The functional connectivity with the supplementary motor area, precentral and postcentral gyri, and prefrontal cortex negatively correlated with tremor scores. These observations confirm the importance of the cerebello-thalamo-cortical pathway in tremor genesis. These, from imaging studies, favor the present hypothesis that a pathological synchronization of IO neurons sparks a chain reaction in the cerebello-cerebral circuits (e.g., synchronous CS, rebound potentiation of DN neurons, and finally rhythmical activation of M1 through the cerebello-thalamo-cortical pathway) (see section Failure of the Short Loop Results in Regular Oscillatory Tremors). In the systematic literature search by Ceresa–Quattrone, who combined the terms ET with the following keywords MRI, VBM, MRS, DTI, fMRI, PET, and SPECT, a total of 51 neuroimaging studies met search criteria, divided into 19 structural and 32 functional studies (93). The studies showed similar findings but without defining a clear topography of the neurodegenerative process. The majority of studies identified functional and structural abnormalities in several portions of the anterior and posterior cerebellar lobules, but the authors stressed the absence of correlation between these neural changes and the clinical symptoms of ET. The authors also highlighted the high variability in results.

We did not expand here on the numerous MRI reports describing the location of lesions in the G–M triangle and the involvement of the central tegmental tract, the dentatorubrothalamic tract, the transaxonal degeneration, and Wallerian degeneration [see the recent work of Raeder et al. (94) focusing on imaging characteristics of transaxonal degenerations involving cerebellar connections].

## Conclusion

We tried to explain complex phenotypes of tremors or tremor-like movements with two physiological principles related to the G–M triangle, pointing out the abnormal motor behavior on the basis of errors in feedforward and feedback loops. The G–M triangle appears in our view as an interface between sensory and motor processes. Tremor is viewed as the result of errors in predictions executed by the posterior fossa structures including the cerebellum, causing an unstable state. Although our hypothesis may not cover all tremors or tremor-like movement disorders, our approach integrates the latest theories of cerebellar physiology and provides explanations how various lesions in or around the G–M triangle results in tremors or tremor-like movements. These two elemental mechanisms can be extrapolated to the loops between dentate nuclei and reticular nuclei in the brainstem acting as reverberation (95). We did not speculate on the neurobiological mechanisms underlying the aberrant synaptogenesis in the G–M triangle (96).

## Data Availability Statement

The original contributions presented in the study are included in the article/supplementary material, further inquiries can be directed to the corresponding author/s.

## Author Contributions

All authors listed have made a substantial, direct and intellectual contribution to the work, and approved it for publication.

## Conflict of Interest

The authors declare that the research was conducted in the absence of any commercial or financial relationships that could be construed as a potential conflict of interest.
